# Autistic Spectrum Disorder in Young People Presenting to a Paediatric Specialist Fatigue Service

**DOI:** 10.1192/bjo.2024.552

**Published:** 2024-08-01

**Authors:** Rachel Chute, Manuela Barona

**Affiliations:** 1 Royal United Hospital, Bath, United Kingdom; 2 University of Bath, Bath, United Kingdom

## Abstract

**Aims:**

To investigate whether young people referred to a paediatric specialist fatigue service present with higher levels of autistic traits or have higher prevalence of Autistic Spectrum Disorders (ASD), than those found in the general population.

**Methods:**

143 initial assessment reports of young people presenting to a paediatric specialist fatigue service were audited over a 5-month period to identify cases where a previous diagnosis of ASD has been documented, or the assessing clinician has recommended referral for an ASD assessment, or autistic traits have been documented in neurodevelopmental screening. Comparative data on age, gender, age of symptom onset, duration of symptoms, reported symptoms, comorbidity, family history, and sleep difficulties was then explored to help us identify/understand the profile of the young people who present to our service. Routine mental health screening questionnaire data from the Revised Children's Anxiety and Depression Scale (RCADS) was analysed in addition to clinical reports regarding mental health comorbidities.

**Results:**

Of the 143 young people presenting to the specialist fatigue service over the 5-month period, 16 had a diagnosis of ASD, and 41 were suspected as having ASD. In total, 39% of service users had, or were suspected of having, ASD. The prevalence was higher in female service users than males with a total of 48% of female service users having, or being suspecting of having ASD, compared with 22% of males. Comparative data demonstrated that autistic/suspected autistic young people presenting to the service were more likely than their neurotypical counterparts to be: over 13 years old, have a longer symptom duration before presentation, have an Educational Health Care Plan, report friendship difficulties, have a family history of neurodiversity, report sensory difficulties, and have sleep difficulties. RCADS scores found that the ASD group were more likely than the neurotypical group to have clinical levels of anxiety (58.3% vs 15.3%) and depression (80.6% vs 58.3%).

**Conclusion:**

Our audit suggests that there is a higher prevalence of young people with ASD/ASD traits presenting to a paediatric fatigue service than found in the general population. Reasons for this may relate to undiagnosed ASD presenting as severe fatigue due to the energy draining nature of camouflaging, as well as sensory overload, known as autistic burnout. Do we need to develop a specialist treatment pathway which is better adapted to these young people's needs? We are planning a follow up study and focus groups to explore this complexity further.

